# Improving health in the Arctic region through safe and affordable access to household running water and sewer services: an Arctic Council initiative

**DOI:** 10.3402/ijch.v75.31149

**Published:** 2016-04-29

**Authors:** Thomas W. Hennessy, Jonathan M. Bressler

**Affiliations:** 1 Arctic Investigations Program, National Center for Emerging and Zoonotic Infectious Diseases, Centers for Disease Control and Prevention (CDC), Anchorage, AK, USA; 2 Arctic Human Health Experts Group, Sustainable Development Working Group, Arctic Council; 3 Section of Epidemiology, Division of Public Health, Alaska Department of Health and Social Services, Anchorage, AK, USA; 4 Applied Epidemiology Fellow, Council of State and Territorial Epidemiologists, Atlanta, GA, USA

**Keywords:** Arctic Council, water, sanitation, infectious, disease, development

## Abstract

Important health disparities have been documented among the peoples of the Arctic and subarctic, including those related to limited access to in-home improved drinking water and sanitation services. Although improving water, sanitation and hygiene (WASH) has been a focus of the United Nations for decades, the Arctic region has received little attention in this regard. A growing body of evidence highlights inequalities across the region for the availability of in-home drinking WASH services and for health indicators associated with these services. In this review, we highlight relevant data and describe an initiative through the Arctic Council's Sustainable Development Working Group to characterize the extent of WASH services in Arctic nations, the related health indicators and climate-related vulnerabilities to WASH services. With this as a baseline, efforts to build collaborations across the Arctic will be undertaken to promote innovations that can extend the benefits of water and sanitation services to all residents.

In 2010, the United Nations General Assembly resolved that access to clean drinking water and sanitation facilities is a basic human right and is essential prerequisites for other human rights (1). The United Nations Millennium Development Goal (UN MDG) #7 (Ensure Environmental Sustainability) has tracked progress towards provision of improved drinking water and showed excellent or good progress in 7 of 9 targeted regions. Similarly, good or excellent progress in provision of sanitation was noted in 6 of 9 regions (2). Because billions of people still lack basic water and sanitation services, the new UN Sustainable Development Goals (SDG) have extended the MDG effort and seek to solve the inequities by 2030 (3), including Goal 6 for universal access to safe and affordable drinking water and adequate sanitation and hygiene for all.

It is understandable that these development efforts have focused on the world's least developed regions and that progress reports use geographical groupings that align with the political structure of the UN. Unfortunately, for those interested in the Arctic region, the MDG reports and other data sources are of limited value for understanding the status of water and sanitation in the circumpolar north. The available data are either separated by national political boundaries or, in the case of the MDGs, are lacking for the region as a whole. The 8 Arctic nations are all considered developed, but several of these countries have a wide range of water, sanitation and hygiene (WASH) infrastructure and are facing considerable development challenges for water and sanitation, especially in rural and remote communities. These local or regional service inequities are lost in the overall picture of large nations with relatively small Arctic populations. For example, a principal tool for measuring water and sanitation services is the WHO/UNICEF Joint Monitoring Programme on Water Supply and Sanitation (4). Data for the United States from June 2015 shows an overall estimate of drinking water piped to homes as 99% (97% for rural populations) and sanitation access as 100% (100% for rural populations). However, these national estimates mask the substantial service inequalities in Alaska, where access to piped, in-home water and sanitation services in rural communities is far from complete, despite decades of effort. Similar problems exist for data from Canada, Russia and Greenland where rural Arctic and subarctic WASH deficiencies are lost in the overall national data, or are lumped into an overall rural category that does not allow for understanding regional trends or deficiencies.

## Health implications of water quality and water quantity

Although the relationship between a safe, plentiful water supply and health is well recognized, the historic focus of public health related to water service has been to prevent diarrhoeal illnesses due to microbiologic contamination of drinking water. Generations of public health professionals have been trained using these famous epidemic events such as John Snow's investigation of the 1854 London Cholera Epidemic (5), the massive outbreak of cryptosporidiosis in Milwaukee, Wisconsin in 1993 (6), or the 2010 Haiti cholera epidemic (7). While preventing such waterborne infections remains a goal of improved access to high-*quality* water, access to adequate water *quantity* is also important for preventing “water-washed” diseases (8,9). Water-washed diseases are those where personal sanitation practices involving water can interrupt transmission. Examples include trachoma (ocular blindness caused by *Chlamydia trachomatis*), bacterial skin infections (*Staphylococcus aureus* furunculitis) and respiratory infections (respiratory syncytial virus bronchiolitis). A growing body of literature has demonstrated that rates of water-washed diseases can be reduced substantially by the provision of adequate water supplies and education efforts to promote hygiene practices (10–13). In Arctic and subarctic communities, households that lack water and sanitation service must self-haul water and then remove human waste in plastic containers commonly called “honey buckets.” This is a time-consuming process which results in contact with raw sewage and has the potential for contamination of drinking water or household surfaces. Furthermore, the physical demands of self-hauling water and limited indoor water storage capacity result in extremely low volumes of water available for household use. For example, in a study of rural Alaska homes without in-home water service, the mean water use was 5.7 L per person per day. This amount would be categorized as a “very high health concern” using WHO standards and is less than the 15 L/person/day recommended for disaster response situations, such as refugee camps (14,15). Such limited access leads to water rationing where priority is given to drinking and cooking at the expense of hand and body hygiene or cleaning of indoor environmental surfaces (16). Limited access to household water is often found in homes with extreme crowding and many young children; these conditions favour transmission of water-washed infections and help to explain the high disease rates seen in many Arctic and subarctic communities.

## Water and sanitation services in the Arctic region

**Fig. 1:**
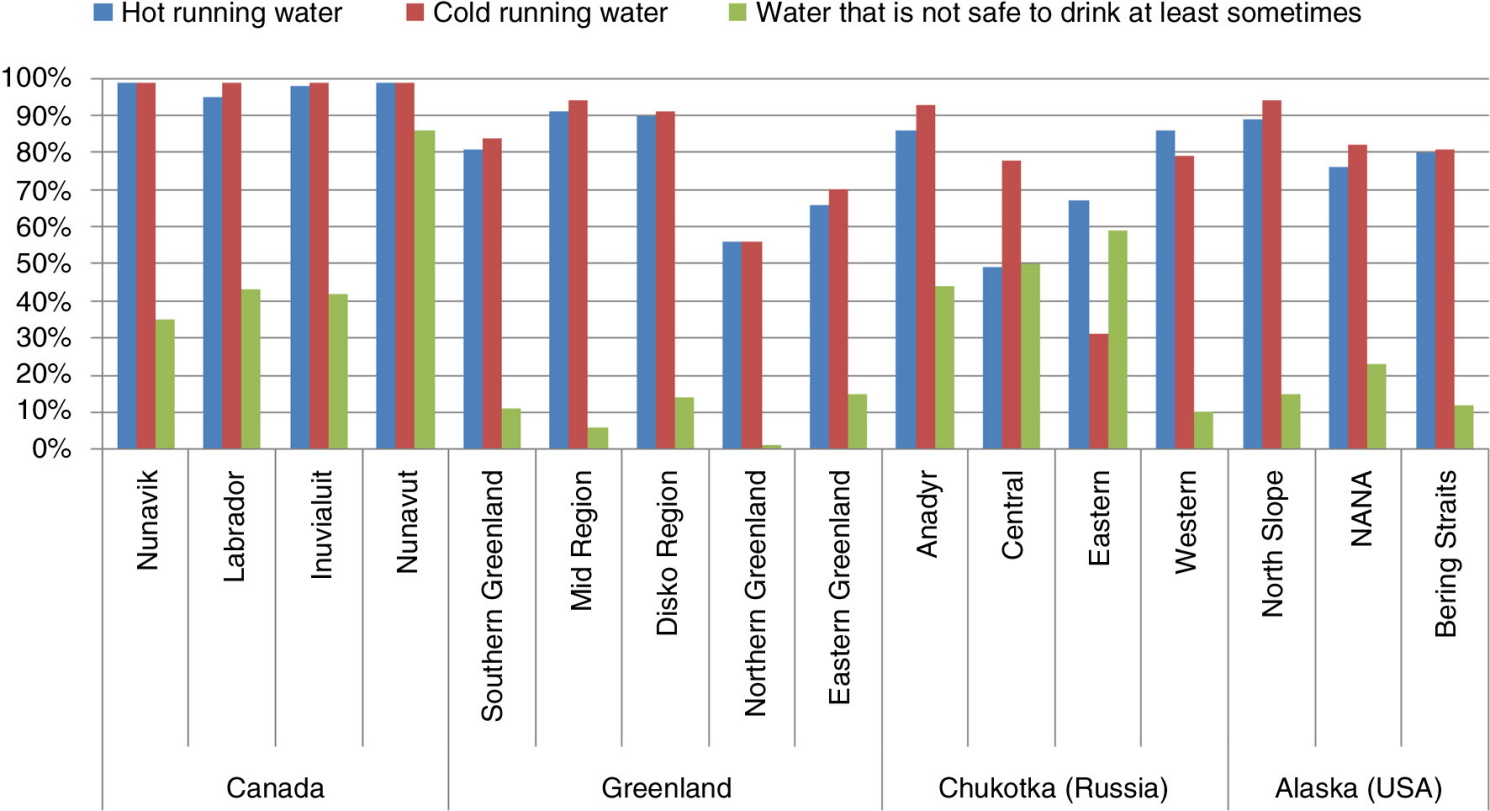
In-home water service in the Arctic by sample community, 2001–2006, Survey of Living Conditions in the Arctic.

Currently, the status of water and sanitation services among Arctic and subarctic populations is not well documented. From 2001 to 2006, the Survey of Living Conditions in the Arctic (SLiCA) was conducted to understand the living conditions in the region, particularly among indigenous peoples (17). This study quantified in-home water access in several areas of the Arctic, showing that access to water services is far from universal. As shown in Fig. 1, in-home access to cold running water ranged from 56% in Northern Greenland to over 99% in all areas surveyed in Canada. Access to hot running water ranged from 49% in Central Chukotka, Russia, to over 99% in Nunavik, Quebec, Canada. In the areas surveyed, having water that was sometimes unsafe to drink ranged from 1% in Northern Greenland to 86% in Nunavut.

**Fig. 2:**
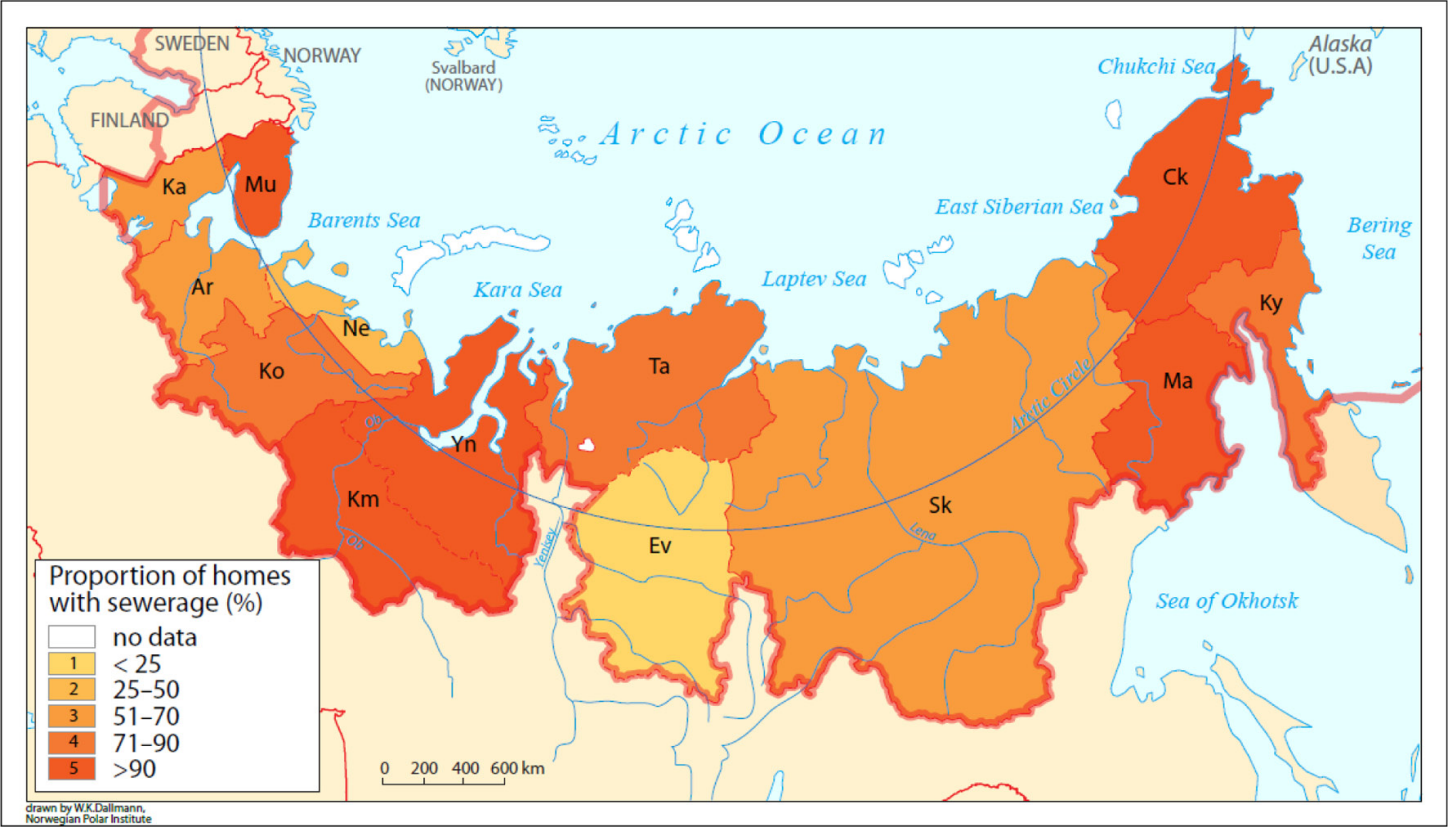
Proportion of homes in Arctic Russian regions with sewerage installation, 2006/2007 NHDP for Russia.

Another data source, the 2006/2007 National Human Development Report (NHDP), showed very low proportions of the population with household sewerage services across large areas of the Arctic and subarctic Russia (Fig. 2) (18). Although useful, the SLiCA surveys and NHDP sewerage survey for Russia were conducted over 10 years ago. Furthermore, SLiCA was limited to Arctic indigenous communities and therefore excluded subarctic populations where access to adequate water and sanitation services is known to be a problem.

The US Census American Housing Survey (AHS) provides ongoing data collection on characteristics of homes using a statistical sampling methodology (19). These data show that, on average during 2010–2014, 69% of rural housing units in the Alaska Native Village Statistical Areas (ANVSA) had complete plumbing facilities, defined as running water to a sink, a flush toilet and a shower or bath. This level of water and sanitation service remains far below the 95.6% of all Alaskan homes and the 99.6% of homes in the overall US population with complete plumbing. While the AHS provides useful data for evaluating trends, interpretation is limited by the sampling frame, where not all communities are included yearly, and the resulting wide confidence intervals around the estimates. The AHS estimates differ from other sources such as Healthy Alaskans 2020, which determined that in 2010, 78% of rural Alaska housing units had in-home water and sewer services (20). The differing estimates in WASH services lead to confusion about the extent of the problem and highlight the need for a comprehensive and updated picture of water and sanitation services in the Arctic region. This would improve our understanding of progress towards the SDG for WASH and allow for comparisons within the circumpolar region.

## Water-related infectious diseases in the Arctic region

Data on waterborne infectious diseases and outbreaks are typically reported by public health authorities in Arctic nations. For example, Dudarev et al. described the water quality and incidence of reportable waterborne disease in the Russian Arctic, Siberia and Far East from 2000 to 2011 (21). Despite data limitations, drinking water quality was found to be very poor with considerable contamination by chemical and biological agents, and high rates of waterborne infectious diseases were observed in these areas. In another paper, Dudarev et al. highlighted large variations in daily per capita water use, which is an indication of water access (22). The authors call for an international collaboration to address water security and infectious disease issues, which they think will be exacerbated by the effects of a changing climate. For the United States, waterborne infectious disease outbreaks are monitored and reported regularly (23).

In contrast to waterborne infectious diseases, water-washed infectious diseases are typically not reported and tracked. This makes documentation of water-related disease rates difficult as special effort is needed to collect and analyse rates of water-washed infections. However, several studies have shown that increased access to water and sanitation services in the Arctic is associated with reduced risk of water-washed infectious diseases. In Alaska, lower rates of hospitalization or ambulatory care visits for respiratory and skin infections is associated with increasing in-home water service among rural Alaska Natives (24,25). Infant hospitalization rates for lower respiratory tract infections (LRTI) in this population are the highest in the United States – 5 times higher for all LRTI than for the general US infant population and 11 times higher for documented pneumonia. In contrast, the diarrhoeal hospitalization rate was similar to that among the general US population. This apparent paradox is likely because safe drinking water is provided in nearly all villages, either by piped distribution or at a community watering point even in villages without piped service. Diarrhoeal infections caused by waterborne diseases are uncommon because this water is safe to drink, but inadequate quantity of water, leading to water rationing, results in higher LRTIs.

Serious bacterial infections are also water-washed diseases. Wenger et al. showed that children who lacked in-home piped water had higher rates of invasive pneumococcal infections (meningitis, bacteraemia) than similar rural children with such in-home service, even after controlling for household income and day-care attendance (26). A recent prospective study of 4 Alaska villages that transitioned from self-hauled water and honey buckets to in-home running water and sewer service demonstrated decreased clinic visits for diarrhoea, respiratory disease and skin infections after installation of running water (27). Another disease entity that could be considered water-washed is dental caries, as access to in-home running water may improve tooth brushing and optimally fluoridated drinking water is associated with lower prevalence of decay in rural Arctic children (28). Although these studies indicate that the burden of waterborne and water-washed infections is higher among persons living without in-home water and sanitation facilities, they do not provide a full view of the health disparities associated with incomplete WASH services across the circumpolar north. A more complete picture could help establish the true burden of excess illness borne by underserved communities and help planners prioritize interventions such as construction, increased health services or prevention strategies such as water fluoridation, education and immunization.

## Climate change threats to water and sanitation services

A complicating factor for the relationship between WASH services and health status is the rapid pace of climate-related change affecting the circumpolar north. Predicted and observed changes in the natural environment could have substantial negative effects on the quality and quantity of source water, the capacity to produce treated drinking water, community distribution systems for water, human waste collection, disposal and treatment. Many of these challenges have been documented (29). For example, worsening erosion and storm surge in the Alaska coastal community of Kivalina led to damage and closure to the community public shower, laundry and toilet system (30). Following this closure, clinic visits for skin and soft tissue infections increased. Changes in source water quality due to increased organic and sediment loads have been documented to result from permafrost-containing river banks melting and subsequently slumping into the river. Permafrost melting and subsequent drainage of tundra ponds have resulted in loss of reliable source water, while rising sea levels threaten some communities with saltwater intrusion into wells used for drinking water (31). In communities built on permafrost, water and sewer distribution lines are commonly contained above ground and insulated against freezing. Permafrost melting can disrupt these distribution systems and have caused some communities to return to self-hauling water and human waste (26). These are just a few examples of what is likely to be an increasing problem in a changing Arctic: community-level challenges that threaten WASH services and increase the risk of waterborne and water-washed human illnesses. There is a need to document these threats to WASH infrastructure in a comprehensive way, along with the community responses and successful adaptation strategies to these changes. This will aid communities and governments to assess risk and develop solutions to preserve WASH services and protect health.

## Assessing the current status of water/sanitation services and health through the Arctic Council

A current, overall picture of the status of WASH services, related health indicators and climate-related threats to these services is lacking for the Arctic. Without such information, regional progress towards the SDG water goals will be hard to assess. Also, without a summary of regional data, we miss opportunities to compare different approaches towards improved health status and to develop best practices that fit the unique challenges of the North. One means of assessing the status of water and sanitation services in the circumpolar north is through the Arctic Council (AC). The AC Sustainable Development Working Group (SDWG) is charged with promoting collaborations to improve the well-being of Arctic inhabitants, and the Arctic Human Health Experts Group (AHHEG) provides support to this effort by undertaking health-related research or initiatives (32). AHHEG includes representatives from each of the 8 Arctic nations and the 6 Permanent Participant organizations. The SDWG has endorsed a project entitled “Improving Health through Safe and Affordable Access to Household Running Water and Sewer in Arctic and Sub-Arctic communities.” The project has 3 objectives: (a) promoting innovations in water and sewer technologies and service provision, (b) documenting the status of water and sewer service and associated health outcomes, (c) describing climate-related vulnerabilities and adaptation strategies related to community water and sewer systems, including source water protection.

The first objective will be met through 2 international conferences. The first will be the ARTEK 2016 Event: “Sanitation in Cold Climate Regions,” which will be held in Sisimiut, Greenland, during 12–14 April 2016, and sponsored by the Danish Technical University, the Arctic Technology Centre and Qeqqata Kommunia (www.conferencemanager.dk/ArtekEvent2016/the-event—background-and-topics.html). The second conference, “Water Innovations for Healthy Arctic Homes (WIHAH),” will be held in Anchorage, Alaska, 18–22 Sep 2016, and sponsored by the State of Alaska and the US Government (www.wihah2016.com/). The WIHAH conference will feature innovative approaches to water and sanitation service that will make such service more affordable, sustainable and available to more homes. The conference will also feature health aspects of water and sanitation, plus approaches to operations, maintenance, policies and regulations that could extend the benefits of water/sanitation services and help the Arctic region meet the SDG by 2030. The second and third objectives will be accomplished through a survey of Arctic region health professionals, water/sanitation engineers, government authorities and community members. The survey can be accessed here: (www.surveymonkey.com/r/arctic_council_water_sanitation) and the preliminary results will be presented at the WIHAH conference, with a final report to the AC in 2017. We encourage interested readers to participate in the survey and to consider attending the International Conferences. These actions will help to build collaborations and to share information across the Arctic that can help create a future where all residents enjoy the health benefits associated with access to in-home water and sanitation services.
